# Robust sequence alignment using evolutionary rates coupled with an amino acid substitution matrix

**DOI:** 10.1186/s12859-015-0688-8

**Published:** 2015-08-14

**Authors:** Andrew Ndhlovu, Scott Hazelhurst, Pierre M. Durand

**Affiliations:** 1 Evolutionary Medicine Laboratory, Faculty of Health Sciences, University of the Witwatersrand, Johannesburg, South Africa; 2 School of Electrical and Information Engineering, University of the Witwatersrand, Johannesburg, South Africa; 3 Sydney Brenner Institute of Molecular Bioscience, University of the Witwatersrand, Johannesburg, South Africa; 4 Department of Ecology and Evolutionary Biology, University of Arizona, Tucson, AZ 85721 USA; 5 Department of Biodiversity and Conservation Biology, Faculty of Natural Sciences, University of the Western Cape, Private Bag X17, Belville, Cape Town, 7530 South Africa

## Abstract

**Background:**

Selective pressures at the DNA level shape genes into profiles consisting of patterns of rapidly evolving sites and sites withstanding change. These profiles remain detectable even when protein sequences become extensively diverged. A common task in molecular biology is to infer functional, structural or evolutionary relationships by querying a database using an algorithm. However, problems arise when sequence similarity is low. This study presents an algorithm that uses the evolutionary rate at codon sites, the dN/dS (ω) parameter, coupled to a substitution matrix as an alignment metric for detecting distantly related proteins. The algorithm, called BLOSUM-FIRE couples a newer and improved version of the original FIRE (***F***unctional ***I***nference using ***R***ates of ***E***volution) algorithm with an amino acid substitution matrix in a dynamic scoring function. The enigmatic hepatitis B virus X protein was used as a test case for BLOSUM-FIRE and its associated database EvoDB.

**Results:**

The evolutionary rate based approach was coupled with a conventional BLOSUM substitution matrix. The two approaches are combined in a dynamic scoring function, which uses the selective pressure to score aligned residues. The dynamic scoring function is based on a coupled additive approach that scores aligned sites based on the level of conservation inferred from the ω values. Evaluation of the accuracy of this new implementation, BLOSUM-FIRE, using MAFFT alignment as reference alignments has shown that it is more accurate than its predecessor FIRE. Comparison of the alignment quality with widely used algorithms (MUSCLE, T-COFFEE, and CLUSTAL Omega) revealed that the BLOSUM-FIRE algorithm performs as well as conventional algorithms. Its main strength lies in that it provides greater potential for aligning divergent sequences and addresses the problem of low specificity inherent in the original FIRE algorithm. The utility of this algorithm is demonstrated using the Hepatitis B virus X (HBx) protein, a protein of unknown function, as a test case.

**Conclusion:**

This study describes the utility of an evolutionary rate based approach coupled to the BLOSUM62 amino acid substitution matrix in inferring protein domain function. We demonstrate that such an approach is robust and performs as well as an array of conventional algorithms.

## Background

The initial steps when investigating phylogenetic relationships or protein functions usually relies on performing accurate sequence alignments. Typically, tools such as BLAST [1] are employed to search a biological database like GenBank [2]. Once a statistically significant match has been made with a protein of known function, hypotheses concerning the putative function or evolutionary history [3] can then be generated for the query sequence. Challenges arise when sequence similarity is low. Conventional alignment approaches are not sufficiently robust to detect homology in rapidly evolving sequences, evolutionary distant organisms or in sequences that have nucleotide and amino acid biases. Sequences may share important functional and evolutionary relationships in the range of low similarity, for example in the region of 20 to 30 % (the “twilight zone” of sequence alignment [4, 5]) or even when similarities are as low as 15 % [6, 7]. Structural data are frequently used as the standard of truth in these circumstances, however, they are often challenging to perform computationally and limited [8]. In the absence of structural data, amino acid residue match or percentage identity based performance measures are used in comparing algorithm alignment quality performances. However, residue based performance measures are flawed [9] and biased as they miss similarities that can only be detected by structural approaches or evolutionary based approaches.

Selective pressure is used to indicate the effects of natural selection on genes and can be used as an indicator of molecular evolution [10]. Evolutionary pressures resulting from natural selection at the DNA level have been found to mould genes into patterns of sites that are highly conserved (resistant to change) and those that are poorly conserved (evolving rapidly). The level of conservation can be inferred from the ratio of the non-synonymous substitution rate (dN or Ka) to the synonymous substitution rate (dS or Ks) and corrected for opportunity, loosely referred to as the evolutionary rate, represented by the parameter ω or dN/dS [11]. It has been demonstrated that these patterns of evolutionary rates or “Evolutionary fingerprints” [12] can be used as a similarity metric in a sequence alignment algorithm. A novel alignment algorithm ***F***unctional ***I***nference using ***R***ates of ***E***volution (FIRE) was developed to address the low similarity challenge [13]. FIRE uses the evolutionary rate (ω = dN/dS) at codon sites, rather than individual amino acid residue identities to align sequences thus circumventing the problem of low sequence similarity. FIRE alignments provided a proof of concept that the evolutionary rate could be used as an alignment metric and that in some cases at least, sequences with similar selection pressure profiles at codon sites have functional similarity. These findings supported the hypothesis that protein domains under similar selective pressures measured through the evolutionary rate may be responsible for similar functions. It has also been suggested that the distribution of the evolutionary rates on a gene could be used in an approach that is analogous to homology searching using a query sequence [12]. Aligning sequences based on their evolutionary rate profiles could therefore offer an additional method for testing functional and evolutionary relationships. One of the major limitations of the FIRE approach, however, was the finding of numerous false positives, particularly when two unrelated highly conserved domains were aligned [13]. This study evaluates the current version of the algorithm (FIRE) and describes the implementation and evaluation of the new, more robust algorithm, which we call BLOSUM-FIRE.

In this study, the evolutionary rate was coupled with a standard BLOSUM substitution matrix in a dynamic scoring function. In doing so the problem of false positives was addressed. The new algorithm (BLOSUM-FIRE) performs as well as MAFFT, T-COFFEE, MUSCLE and CLUSTAL Omega algorithms. An evolutionary rates database (EvoDB) is reported and described elsewhere (manuscript accepted in *Database*) and can be queried with FIRE data. As a test case, the enigmatic hepatitis B virus X protein (HBx) was examined with BLOSUM-FIRE. The Hepatitis B Virus (HBV) has been implicated in diseases such as Hepatocellular carcinoma (HCC) [14], chronic hepatitis and liver cirrhosis affecting millions of people worldwide. One of the challenges in understanding the biology of the HBV has been the failure to elucidate the numerous functions of the HBx protein [15]. Experiments have failed to conclusively identify the role played by the protein in the hepadnavirus life cycle [16]. Consequently, its structure and function has sparked controversy and speculation. This controversy is attributed to a lack of homology to any known protein in biological databases, exacerbated by the fact that the structure has defied conventional structure determination methods [17]. Here we provide alignment evidence that the protein may harbour viral endopeptidase functions.

## Methods

### The FIRE algorithm

The FIRE algorithm is implemented in the Python programming language, it finds the optimal alignment of two amino acid sequences using the Bayes Empirical Bayes (BEB) maximum likelihood estimates (MLEs) [11] of the evolutionary rate (ω = dN/dS) at codon sites. FIRE requires two multiple sequence alignments (MSAs) of nucleotide sequences with their corresponding phylogenetic trees to generate a pairwise amino acid alignment. A modified Needleman-Wunsch algorithm [18] determines the global alignment between the sequences based on a dynamic programming (DP) approach. The generation of alignments requires MSA files and their corresponding phylogenetic tree files which are used as input for the CODEML program found in the Phylogenetic Analysis by Maximum Likelihood (PAML) suite of software [19] to produce the BEB MLEs of ω at codon sites. This pre-processing using CODEML provides the *rst* output files which are consequently used as input for the FIRE algorithm to generate alignments.

### Data sets

For the initial evaluation of the algorithm, data sets used in the concept paper were utilised [13]. To evaluate the BLOSUM-FIRE algorithm evolutionary rate profiles of the Pfam (Protein family) [20] database were compiled into the evolutionary rates database (EvoDB) described elsewhere (manuscript accepted in *Database*).

To demonstrate the utility of the database evolutionary profiles of the enigmatic HBx protein were used. HBx was chosen because functional inference has been elusive. HBx curated nucleotide sequences were obtained from [21]. An alignment of 20 nucleotide sequences of the HBx was generated using the CLUSTAL Omega (ver. 1.2.0) program and phylogenetic trees inferred from the FastTree program (ver. 2.1.7) [22]. MLEs of ω were determined using the CODEML program in PAML (ver. 4.4) suite of software.

To infer the domain functions of HBx the EvoDB database was used. EvoDB (www.bioinf.wits.ac.za/software/fire/evodb) is a database of 98 % of the gapped nucleotide sequence alignments for the PFAM-A database. It provides the evolutionary rate (ω = dN/dS) profiles determined under the M2a model (CODEML algorithm in the PAML suite) for 97 % of the PFAM domains. The clustering of proteins into families in PFAM using domain functions provided a suitable framework for implementing a searchable database for inferring domain functions. The database was compiled for use by BLOSUM-FIRE. Briefly, BEBs [11] ω MLEs at codon sites were calculated using the CODEML program (PAML ver. 4.4) [19] under the M2a Model (*NSsites = 2)*. This parameter assumes one ratio for all the branches and allows for the detection of positive selection at codon sites. The ω MLEs were extracted from the *rst* CODEML output file and used to compile the ω MLE profiles for each dataset. This *rst* file contains supplemental results including: Naive Empirical Bayes (NEB) probabilities for the site classes of ω, a list of positively selected sites, log likelihood values and the BEBs. These *rst* files for PFAM domain analysis using CODEML under the M1a and M2a models are available for download on EvoDB. Individual domains form functional units and are less variable than multi-domain proteins. The evolutionary rates at codon sites across discrete domains provide signature profiles of ω values, which may be used for homology detection [13]. An independent study of RNA viruses based on a novel model of sequence evolution also demonstrated that protein-coding regions were moulded into sites of varying selective pressure [12].

To evaluate the accuracy of the new BLOSUM-FIRE algorithm against widely used algorithms: CLUSTAL Omega, MUSCLE and T-COFFEE using MAFFT as a reference, 20 datasets were used. The datasets consisted of ω profiles of unrelated and related domains using nucleotide sequence data for the PFAM-A domains obtained from EvoDB.

### Evaluation of the FIRE algorithm with real data

The limitation of the FIRE algorithm identified in the concept paper of [13] was that the algorithm produced false positive results in certain datasets . Those data sets where the FIRE algorithm produces false positives were identified, analysed and the correlation between the statistical distribution of the ω values and the quality of alignments produced was investigated. To further explore the problem posed by false positives, 100 alignments of evolutionary rate profiles of the PFAM-A database were investigated. Alignments were generated using the FIRE algorithm and the number of residues aligned was counted as a measure of the quality of alignment. Alignments were then generated using the CLUSTAL Omega algorithm (ver. 1.2.0) [23] and MAFFT (ver. 7.130b) algorithm [24] using default parameters. Each alignment was scored using the numbers of aligned residues normalised by maximum sequence length indicated by the identity score. Our identity score is equivalent to the percentage identity score (PID) [9] normalised between 0 and 1 for intuitive comparison to a FIRE algorithm score. To assess the false positive rate we defined a false positive as any alignment with a FIRE score above 0.6 for functionally unrelated domains. This threshold was adopted from the concept study where domains with this score or higher were inferred to share similar domain functions. Each alignment was scored using the numbers of matched residues normalised by the maximum sequence length indicated by the identity score. In such scenarios, structural data could provide a standard of truth for evaluating alignment quality [25]; however, the requirement for MSAs in BLOSUM-FIRE and the limited availability of domain structures made this impossible in this study.

### Evaluation of FIRE using simulated data

The FIRE algorithm had reduced specificity in highly conserved data sets resulting in false positives. Simulated data sets were therefore created to investigate false positives further. Highly conserved datasets were created such that the ω values were in the range [0,0.02] across all coding sites. Simulations were carried out using real and truncated datasets generated by a combination of custom scripts and manual editing of input files to insert sites of positive selection. Alignments were generated using FIRE default parameters for gap open and gap extension penalties of 0.5 and 0.1, respectively. The simulated datasets were then aligned using the MAFFT and CLUSTAL Omega algorithms.

### Coupling FIRE with a BLOSUM substitution matrix

Evaluation of the false positive results identified in the concept paper [13] revealed that the homogeneity of highly conserved data, where the variance of ω values was low, resulted in reduced specificity. To address this challenge the new algorithm called BLOSUM-FIRE was implemented by incorporating the identity of the amino acids using the BLOSUM62 substitution matrix [26]. Recently, the efficacy of substitution matrices has been questioned with the development of search approaches that do not utilise amino acid substitution matrices, for example, the CS-BLAST tool, is a novel search approach in which context-specific (CS) substitution matrices were incorporated into the BLAST algorithm [27]. At the same time, MIQS (Matrix to Improve Quality in Similarity search), a more robust matrix, has been developed from numerous matrices using principal component analysis for detecting remote homologies, for example, in transmembrane regions [28].

Empirical experiments with simulated data sets provided the framework for coupling FIRE and the BLOSUM62 substitution matrix. We propose a scoring function that scores the aligned amino acids depending on their conservation measured through the ω parameter. To simplify the task of generating alignments using the Needleman-Wunsch DP algorithm, a unified scoring function was required. Therefore, for two amino acids: *i* and *j* with evolutionary rate values of *ω*_*i*_ and *ω*_*j*_, the guiding principles for the BLOSUM-FIRE scoring function are:

- Determine the BLOSUM amino acid score *s*(*i,j*), from the BLOSUM matrix using the identity of the aligned residues.
- Determine the selective pressure on the amino acids using the values of *ω*_*i*_ and *ω*_*j*_.
- Scale the BLOSUM score using the following principles of selective pressure: *ω* > 1 is positive selection, *ω* < 1 is negative selection or purifying selection and *ω* = 1 indicates neutral selection.
- Determine the similarity of *ω*_*i*_ and *ω*_*j*_ values normalised between 0 and 1 to a score called *similarity*.
- Use the selective pressure to scale BLOSUM values to a BLOSUM comparable score called *selection*.
- Combine the *similarity* score and *selection* score to obtain the BLOSUM-FIRE score.
- Use a modified Needleman-Wunsch DP algorithm to obtain the global alignment of the amino acid sequences.

To simplify the scoring function, a single value was required to score the *similarity* of the evolutionary rates (dN/dS) and *selection* of the selective pressure adjusted BLOSUM score. We formulated an additive scoring scheme such that:

$$ BLOSUM\hbox{-} FIRE= similarity+ selection $$

Therefore, the BLOSUM-FIRE scoring function scores amino acid residues using BLOSUM scores which are subsequently adjusted according to selective pressure and this is added to similarity score of ω values aligned at codon sites.

### Approximating selective pressure and scaling the BLOSUM score

The ω value is widely accepted as a proxy for the evolutionary pressure on an amino acid at a codon site. This has been extended to indicate whether the amino acid has a functional role and identifying the coding regions in a genome [29, 30]. An ω value close to zero indicates strong selective pressure, while an *ω* > 1 means non-synonymous substitutions may offer some fitness advantage to the function of the protein concerned at that site. In addition, amino acid variability at such sites with elevated ω values is expected. On the other hand, *ω* <1 indicates negative or purifying selection and *ω* = 1 indicates neutral selection. We propose an approximation equation based on these selective pressure guidelines. This equation was chosen as it is monotonic and, more importantly, it approximates the relationship between ω values and the selective pressure according to theoretical guidelines, for example, detecting sites under positive selection [31]. Let *a* be the ω value of *amino acid*_*i*_ and *b* is the ω value of *amino acid*_*j*_, the approximated selective pressure on the two aligned amino is acids such that:

1 $$ se{l}_{ab}={e}^{-\left(a+b\right)} $$

where *sel*_*ab*_ is the approximated selective pressure at the codon site in the range [0,1]. The selective pressure (*sel*_*ab*_) provides information that is untapped by conventional alignment algorithms. Such an approximation given by Equation (1) emphasises the quality of alignments based on the level of conservation at the amino acid level. We propose that this selective pressure can be used to scale BLOSUM scores. This selective pressure scaled BLOSUM score called *selection* is given by the equation:

2 $$ selection= BLOSUM\times se{l}_{ab} $$

The premise of the FIRE principle is that amino acid sequences under similar selective pressures share similar functions. Several studies have demonstrated that highly conserved sites can be used as a proxy for functionality, for example, ConSurf [32] and Rate4site [33]; it has also been demonstrated that natural selection imprints genes with evolutionary fingerprints which can be used as gene identifiers [12]. Our concept study [13] demonstrated that at least in some cases those domains that are under similar selective pressures inferred from the evolutionary rates can be responsible for similar functions. While amino acid sequences under similar selective pressures may share similar functions the converse is not true. Let *a* be the evolutionary rate (*ω*) of *amino acid*_*i*_ and *b* the ω value of *amino acid*_*j*_, where *i* and *j* are indexes in amino acid sequences, then the similarity *sim*_*ab*_ of the evolutionary rates can be calculated such that:

3 $$ si{m}_{ab}=1-\frac{\left|a-b\right|}{ \max \left(a,b\right)}\kern1em \mathrm{if}\kern0.5em a\kern0.5em  or\kern0.5em b\ \ge\ 1 $$

or

4 $$ si{m}_{ab}=1-\left|a-b\right|\kern1em \mathrm{if}\kern0.5em a\kern0.5em  and\kern0.5em b<1 $$

Either Equation (3) or Equation (4) can be used to calculate the similarity *sim*_*ab*_ to the absolute difference normalised in the range [0,1]. Therefore, *sim*_*ab*_, maximises the absolute normalised differences at codon sites.

### Coupling the BLOSUM matrix approach with the FIRE approach

To couple the two approaches an assumption that an amino acid match is analogous to the similarity of low evolutionary rates at a highly conserved site was adopted. Therefore, an identical match score in amino acid residues was proposed to be equivalent to a FIRE score of 1 for a highly conserved site. A proportionality constant (*K*) was proposed for the FIRE approach score or *sim*_*ab*_ to be comparable to BLOSUM scores. Let *sim*_*ab*_ from Equation (3) or (4), be the similarity between the ω values at sites *a* and *b*, therefore the BLOSUM comparable score is given by:

5 $$ F=K\times si{m}_{ab} $$

where *F* is a BLOSUM comparable score, *K* is the proportionality constant obtained from the substitution matrix and *sim*_*ab*_ is described in Equations (3) and (4). To calculate *K*, an assumption that an identical amino acid match is analogous to a match at codon sites where the evolutionary rates are close to zero was adopted. The proportionality constant (*K*) is the mean amino acid identical match scores determined from the BLOSUM62 matrix and it was found that K = 5.64. Using this proportionality framework we adopt the BLOSUM approach and weigh ambiguity characters in the same manner as regular amino acids.

### The BLOSUM-FIRE scoring function

The strength of the FIRE approach is its ability to detect shared functions using ω values at low sequence similarity without using the amino acid identity. The BLOSUM approach is effective for aligning sequences using amino acid identity. The FIRE algorithm produced false positives when sequence conservation was high. This is because at high conservation the high similarity of the ω values creates “noise” for the scoring function resulting in poor alignments. Under such conditions the power of aligning sequences based on dN/dS values at amino acid sites becomes weak. Therefore, at high conservation the BLOSUM score is more acceptable than a FIRE score.

To allow the algorithm to be dynamic between the two approaches: BLOSUM to FIRE depending on the selective pressure on the amino acids the equations were combined, resulting in a dynamic scoring scheme such that:

6 $$ BLOSUM\hbox{-} FIRE= BLOSUM\times se{l}_{ab}+K\times si{m}_{ab}\left(1-se{l}_{ab}\right) $$

Conversely, the approach recognises that when conservation is low the BLOSUM score may not be strong evidence. It has not left our attention that determining evolutionary rates requires nucleotide MSAs, which can be challenging to obtain. Therefore, to mitigate this challenge the database EvoDB is provided (described above) and BLOSUM-FIRE allows for the comparison of protein sequences with evolutionary rate profiles in a one sided comparison. However, this comparison may not be as robust as a pairwise evolutionary rates comparison. To allow for this one-sided comparison, the algorithm assumes that all the protein residue sites for the protein sequence without ω MLEs are highly conserved such that ω = 0.

### A variable gap penalty scheme

The affine gap penalty scheme used in the FIRE algorithm proposed by [34] and [35] was modified for the BLOSUM-FIRE algorithm to make it more variable depending on the selective pressure at codon sites. A variable gap penalty scheme similar to the form described by [36] is proposed. Furthermore, Equation (1) was also adopted for approximating the selective pressure at codon sites to determine the gap penalties. The proposed variable gap penalty uses the same principle for adjusting BLOSUM scores. Consequently, those sites that are highly conserved have relatively high gap penalties given by the equation:

7 $$ P=g{e}^{-a}+tl{e}^{-x} $$

where *P* is the total gap open penalty *g*, is the initial gap open penalty *t*, is the initial gap extension penalty *l*, is the length of the gap, *e* ^− *a*^ is the approximated selective pressure at the codon site, *a* is the ω value where the gap is opened and similarly, *e* ^− *x*^ is the approximated selection pressure where *x* is the ω value for the site where the gap is extended. Therefore, gaps are extended based on the level of conservation at each site where the gap open penalty and its extension thereof varies as a function of the evolutionary rate at each site. According to this penalty scheme, variable sites are more likely to be gap insertion or extension sites than conserved sites.

### Determining optimal gap penalties for the new BLOSUM-FIRE algorithm

Theoretical guidelines for the determination of gap penalties are scarce. In this study an empirical approach similar to the investigation by [37] was adopted. The datasets consisted of 50 amino acid sequences with their ω MLEs of varying sequence lengths randomly selected from the Pfam-A seed alignments database (ver. 27.0) [20]. The BLOSUM-FIRE score was used as a proxy for the quality of the alignments, the score is the mean of the sequence identity and the normalised evolutionary rate score (old FIRE score) for that alignment. We aligned the Pfam sequences against each other for iterations of the gap penalties. Iterations were carried out using gap open penalties in the range [0,11] and gap extension penalties in the range [0,6]. Analysis was only carried out on the gap penalties and the BLOSUM-FIRE score for that alignment.

### Evaluating the effect of MSA quality on the final alignment

We evaluated the effect of sequence number and MSA accuracy on the final alignment. To evaluate the effect of number of sequences on the final alignment, 10 Pfam families were randomly selected and using between 3 and 20 taxa we investigated how shuffling the order of sequences in the MSA affected the BLOSUM-FIRE score. The domains with the varied number of sequence were aligned with the same domain with 20 sequences. The effect of MSA accuracy on the final alignment was investigated using the heat shock hsp70 (PF00012) domain using nucleotide sequence data corresponding to the manually curated Pfam alignments from EvoDB. Using the MAFFT algorithm and by iteratively increasing the gap open penalty from 0 to 3 in increments of 0.1, we were able to obtain alignments of varying quality and accuracy using the comparison against the alignment generated using default parameters as a performance measure. These alignments were then aligned to the reference alignment to evaluate the effect on the final alignment. Phylogenetic trees for the above experiments were determined using the CLUSTALO program and ω profiles were determined under the M2a model using CODEML.

### Resources

The resource requirement of an algorithm is an important consideration as some algorithms may require prohibitive resources or running times. While BLOSUM-FIRE has not been optimised for performance, we assessed the time and resource requirements for generating alignments from the MSAs to the final pairwise alignment. For this analysis, the heat shock hsp70 domain was used; we trimmed the sequence to 900 nucleotides to approximate the average length of a protein. Alignments were then de-aligned and iteratively added from 3 sequences to 30. Resources were measured using the Unix *time* command. Execution times were measured for the generation of alignments and calculating the phylogenetic tree and determining the ω MLEs. Furthermore, the time taken to generate the final alignment using BLOSUM-FIRE was also measured. Resources were measured on one of the nodes on our cluster (WITS-CORE). This is an Intel (R) Xeon (R) machine with 15 E5630 processors at 2.53GHz running the Scientific Linux operating system. The machine has a cache size of 12M and 23GB RAM. All experiments were carried out on a single core using default algorithm parameters.

### Evaluation of BLOSUM-FIRE performance

The conventional approach when evaluating the performance of a sequence alignment algorithm is to make use of a sequence alignment benchmark. However, the reliance on pre-processed data makes it a challenge to evaluate the BLOSUM-FIRE approach. The MAFFT algorithm is well known for its accuracy and speed, for example, [38] and more recently [39] and was therefore chosen as a reference aligner for evaluating the new implementation. Datasets comprised 10 unrelated and 10 related domains obtained from EvoDB. To demonstrate the utility of our approach and EvoDB, we simulated the 10 related datasets by randomly selecting and generating an alignment of 10 taxa from the selected families with total sequence numbers in the range [14,187] and these were then aligned against their full alignments. The quality of alignments was measured using the Sum of Pairs Score (SPS) and Total-Column score (TC) implemented in the *bali_score* tool provided with the BAliBASE benchmark database [40]. The SPS is the ratio of number aligned pairs in the test alignment to the number in the reference alignment; it evaluates the quality of the alignment produced. The TC score is a binary score of the comparison of the test and reference alignment for each column and the SPS was measured with MAFFT as the reference alignment.

### The statistical significance of the alignments

To evaluate the significance of the results, a statistical framework was required. The inference of homology requires assessment of the statistical significance of an alignment to identify those real alignments from those due to chance. A *p*-value framework was implemented to assess the statistical significance of the alignments in EvoDB. The HBx ω MLEs were shuffled and used to query the EvoDB database. The scores of the alignments were tested for statistical significance using a *p-*value statistical framework. In this framework, the fraction of shuffled alignments scoring higher than the actual alignment was determined and this provided the *p-*value for that alignment. The functions of those proteins that were statistically significant were assessed by analysing the alignments produced. The BLAST algorithm finds statistically significant regions of local similarity between two sequences. The most statistically significant results found by BLOSUM-FIRE were then assessed using the BLAST algorithm to determine their statistical significance using a local alignment approach.

## Results and discussion

### Evaluation of the FIRE algorithm using real and simulated data

**Fig. 1:**
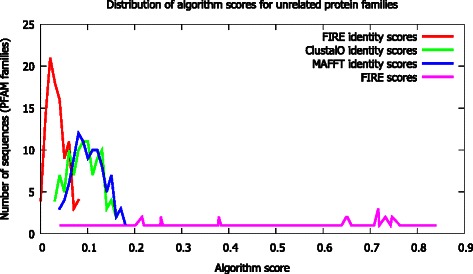
Comparison of the FIRE algorithm identity score distribution with CLUSTAL Omega and MAFFT algorithms for 100 alignments of low sequence similarity. Identity scores were calculated by normalising the number of matched residues to maximum sequence length. The FIRE score is the algorithm score for the aligned sequence based on the evolutionary rate approach. The divergence in identity score distributions between FIRE and the other algorithms reveals that the FIRE algorithm produces poor quality alignments compared to the conventional algorithms

To demonstrate false positive results, 100 random alignments of varying sequence lengths were aligned using FIRE. The quality of alignments was inferred from identity scores. Alignments were then performed using the CLUSTAL Omega and MAFFT algorithms (Fig. 1). The results indicate that the FIRE algorithm scores are relatively high; however the distribution of the identity scores indicates that the alignments are poor. Therefore, the FIRE algorithm had a high rate of false positives (28 %) presented by high algorithm scores with low alignment identity scores and poor alignments compared to the conventional approaches.

**Fig. 2:**
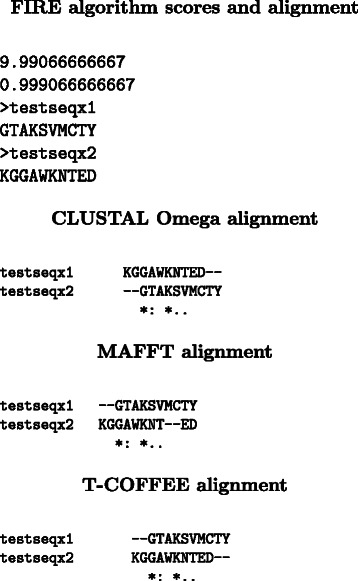
Alignment produced by the FIRE algorithm for simulated highly conserved sequences compared with conventional algorithms: CLUSTAL Omega, MAFFT and T-COFFEE. The first line in the FIRE output indicates the normalised score of ω score matches and the second line is the FIRE score for the alignment, FIRE scores above 0.6 indicate functional similarities in the sequences. The FIRE alignment demonstrates a false positive result

Using highly conserved truncated simulated datasets, the canonical false positive result provided by the FIRE algorithm is provided in Fig. 2 and for comparison the same alignment produced by conventional algorithms. These results demonstrate the false positives challenge of the evolutionary rate (FIRE) approach [13]. The FIRE algorithm has a very high algorithm score for the alignment, however, the alignments are poor. It is important to note that the comparison of quality performance using MAFFT as reference alignments to obtain the SPS or the number of residues matched through an identity score (Fig. 1) are biased, only structural comparison approaches can provide better standards of truths or benchmarks [41]. However, the challenge is that generating the evolutionary rates used for the FIRE algorithm where MSAs are used instead of single sequences means that a structural comparison framework is difficult to implement. This brings to question whether counting the number of matched residues is a valid quality performance measure. Without structural data residue based performance measures such as PID or the identity score (Fig. 1) present the most viable option although their use is not recommended [9]. On the other hand, the aim of this work is to demonstrate that our coupled approach can perform as well as residue based approaches. Numerous benchmarks exist for assessing the performance of algorithms [40], however, these cater for protein sequence analysis, the unique requirement of BLOSUM-FIRE for MLEs of evolutionary rates through CODEML means that these benchmarks cannot be utilised.

### Approximating selective pressure at codon sites and scaling the BLOSUM score

**Fig. 3:**
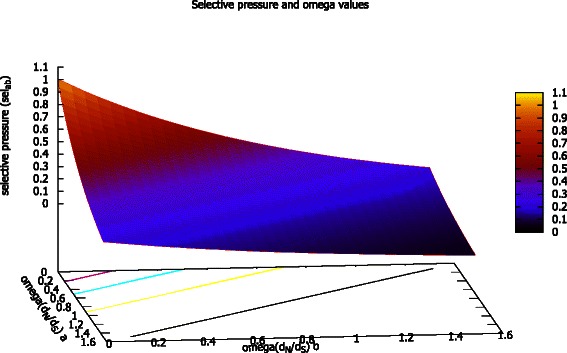
Three dimensional heat map for the approximation of selective pressure (*sel*_*ab*_) using dN/dS values at sites *a* and *b*. The selective pressure decreases as the values of dN/dS increases

We compared the quality performance of the evolutionary rate based approach (FIRE) with the conventional BLOSUM substitution matrix based algorithm. The results of the comparisons provided the theoretical framework required to conceptualise an approach to couple the evolutionary rate based approach with a conventional BLOSUM matrix. Equation (1) was used to approximate the proposed relationship between the total ω values and selective pressure *sel*_*ab*_ at aligned amino acid sites. We looked into exploiting the information provided by the ω parameter as an indicator of selective pressure to scale BLOSUM scores. The selective pressure at codon sites is used to scale the BLOSUM score using Equation (1). Therefore, according to the approximation, the impact of the BLOSUM score decreases with increased ω value as the selective pressure *sel*_*ab*_ on the amino acids decreases. The rationale is that the selective pressure on the codon site decreases as we move from negative selection (*ω* <1) to positive selection (*ω* >1). A three dimensional heat map providing the correlation between ω values: *a* and *b* and the approximated selective pressure *sel*_*ab*_ is provided in Fig. 3.

**Fig. 4:**
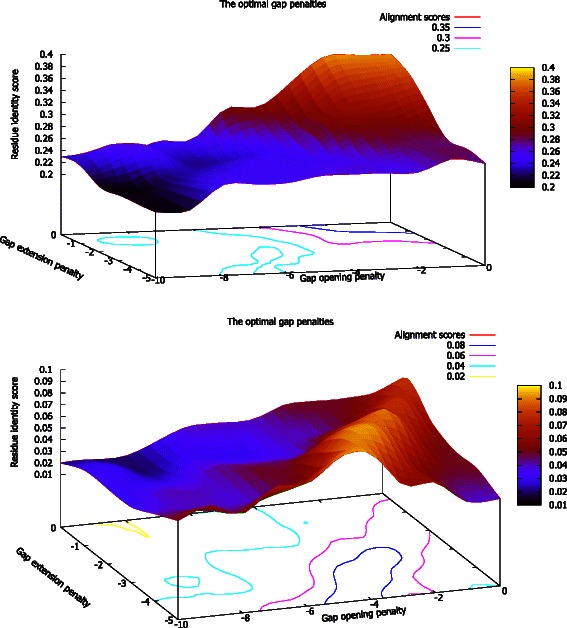
Determining the optimal gap penalties. The plots were produced using the median of the 5 high scores (above) and the median of the 5 low scores (below). The scores were obtained from 6930 alignments from iterating through the gap parameters using an affine gap penalty scheme

To determine optimal gap penalty parameters for BLOSUM-FIRE, alignment scores were obtained from iterations of the gap open penalty from 0 to -11 and gap extension penalties from 0 to -6 were used to generate Fig. 4. The plots show heat maps for the optimal gap open penalty and gap extension penalty for proteins of low sequence similarity. A gap open penalty of -4 and extension penalty of -1 was identified.

### Resources and accuracy

**Fig. 5:**
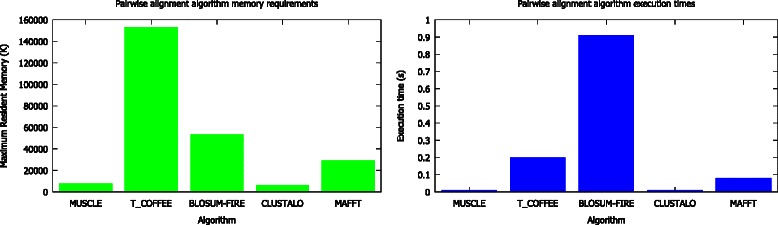
Resource requirements for the BLOSUM-FIRE algorithm and other widely used algorithms. BLOSUM-FIRE is not optimised for performance and has the highest execution times and requires the most memory after T-COFFEE compared to the widely used algorithm. It is important to note that BLOSUM-FIRE is implemented in an interpreted language and the other algorithms are implemented in compiled languages

**Fig. 6:**
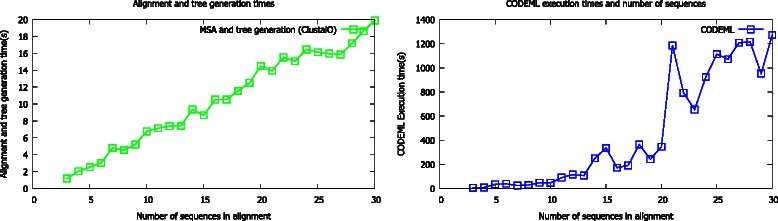
The time requirements for generating alignments using CLUSTALO and determining the evolutionary rate profiles scales with the number of sequences

We compared the resource requirements of our algorithm with other widely used algorithms. The relatively high executive times and resident memory requirements (Fig. 5) for BLOSUM-FIRE are not surprising as it is implemented in Python an interpreted language and widely used algorithms are implemented in compiled languages optimised for performance. At this point we provide BLOSUM FIRE, as a viable alternative to the widely used tools and future versions of BLOSUM-FIRE will be implemented in faster and optimised compiled programming languages. The results for the typical resource requirements (Fig. 6) for the pre-processing showing the times required for the maximum likelihood estimation with CODEML and generation of tree and alignment, in this case using CLUSTALO scales with the number of sequences and maybe prohibitive. To address this challenge we have provided EvoDB and work is in progress to integrate BLOSUM-FIRE into EvoDB to provide a user friendly web interface in a similar form to BLAST and GenBank.

**Fig. 7:**
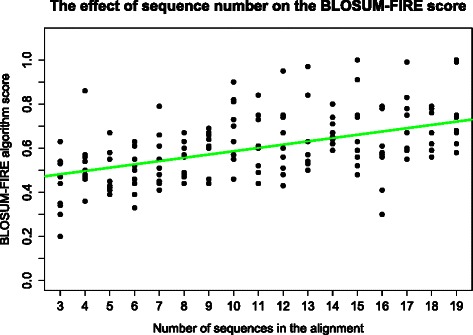
The effect of randomly adding homologous sequences from other taxa and performing an alignment on BLOSUM-FIRE score using CLUSTALO phylogenetic trees

The evaluation of the effect of MSA accuracy (results not shown) on the final alignment revealed that there is a correlation between the accuracy measured through the SPS and TC scores and the BLOSUM-FIRE algorithms score for alignments. Therefore, the choice of alignment must be made appropriately. The evaluation of number of sequences on the final alignment (Fig. 7) revealed that generally adding more homologous sequence results in an increase in the BLOSUM-FIRE score. On the other hand, the results demonstrate the importance of choosing representative taxa to obtain more accurate results as has already been suggested by the work of [42]. The efficacy of our approach relies on the accuracy of input data from MSAs, phylogenetic trees and calculated ω profiles. Anisimova et al. [42] examined the accuracy of detecting sites under positive selection using the CODEML program and found that sequence length had a small effect on the accuracy of the model results. It was also found that the numbers of taxa, size of the tree and sequence divergence were correlated with model accuracy. In line with guidelines in the CODEML documentation a range of 4 to 20 sequences is recommended for analysis. We also suggest monitoring sequence divergence through the dS parameter and recommend stringent total dS in the range [0.1,0.9] and advise caution for those evolutionary rate profiles not meeting this criterion.

### Performance of the BLOSUM-FIRE algorithm

**Fig. 8:**
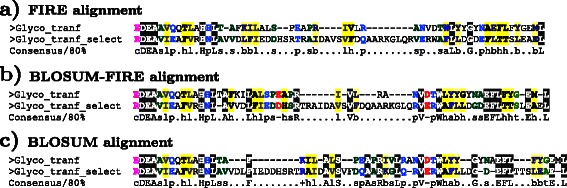
Comparison of alignments for the Glycosyltransferase family 28 N-terminal domain (PF03033.15) using 4 taxa for the Glyco_tranf_select and 36 taxa for the Glyco_tranf. These alignments demonstrate the difference between the BLOSUM-FIRE and the individual approaches used in its conceptualisation

We evaluated the performance of BLOSUM-FIRE by comparison with conventional, widely used algorithms CLUSTAL Omega, MUSCLE and T-COFFEE, using MAFFT reference alignments to obtain the SPS. Additionally, alignments produced by a plain BLOSUM62 based algorithm are provided for comparison to demonstrate the difference between the new algorithm and a standard BLOSUM matrix based algorithm. Figure 8, annotated using CHROMA [43], displays alignments for the Glycosyltransferase domain provided to demonstrate the difference between the individual approaches and the coupled approach. The BLOSUM-FIRE and BLOSUM alignment on its own exhibit differences confirming that the new BLOSUM-FIRE is not swamped by the BLOSUM62 based approach; it does not outweigh the evolutionary rate based approach as the alignments differ from those produced by the BLOSUM based approach. Using a pairwise approach does not take advantage of some the heuristic MSA algorithm features, for example, alignments generated do not benefit from the consistency objective function in the T-COFFEE algorithm. Therefore, our comparison only evaluated the efficacy of aligning residues. At the same time BLOSUM-FIRE is a pairwise sequence alignment algorithm; this makes such a comparison fair.

**Fig. 9:**
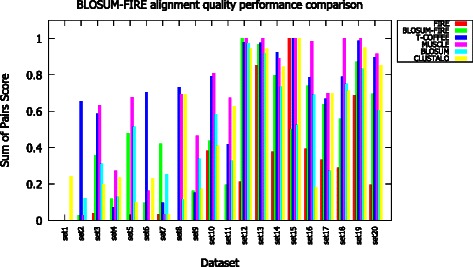
Accuracy of the BLOSUM-FIRE algorithm compared with widely used algorithms and for comparison the FIRE and BLOSUM based algorithm scores. MAFFT alignments were used as references to obtain the Sum of Pairs scores. Datasets (1-10) are unrelated and (11-20) are related

Modifications and improvements to the algorithm were evaluated by comparing the quality performance measured through residue identity scores using datasets of low sequence similarity. The quality performance results (Fig. 9) of BLOSUM-FIRE compared against widely used algorithms is provided. The results reveal that in almost all instances BLOSUM-FIRE performs better than CLUSTALO and FIRE. The results also show that MUSCLE and T-COFFEE have the best accuracies. Comparison of the performances of BLOSUM, FIRE and BLOSUM-FIRE demonstrate that the new implementation is different from its individual approaches: BLOSUM and FIRE. It is important to note that to evaluate the accuracy of BLOSUM-FIRE using MAFFT may not be appropriate or accurate and may be biased as MAFFT is only using residue homology to generate alignments. However, we provide this comparison to show that our algorithm based on this coupled approach has accuracies similar to conventional algorithms.

### EvoDB results for the HBx query

To demonstrate the utility of the BLOSUM-FIRE algorithm, the enigmatic HBx was aligned against all the evolutionary rate profiles on EvoDB (a database of evolutionary profiles for the Pfam-A entries). The evolutionary rate profile of the HBx and EvoDB were determined using the CODEML program (see methods). The top five statistically significant alignments are provided in Table 1. BLOSUM-FIRE was able to accurately identify the HBx family, PF00739.14, as the most statistically significant. Comparison of the old FIRE and BLOSUM-FIRE scores demonstrates how robust the coupled approach is over the FIRE approach. The BLOSUM-FIRE score is the mean of the dN/dS similarity score and the normalised identity score of the alignment. These results also demonstrate the limitations of the evolutionary rate (FIRE) approach scores indicated by the dN/dS *sim* score.

**Table 1:** The top 5 statistically significant results of HBx aligned against EvoDB PFAM-A profiles

PFAM ID	Description of family	BLOSUM-FIRE score	PID	dN/dS *sim*	Residues Matched	*p*-value
PF00739.14	Trans-activation protein X family.	0.62	76	0.49	113	0.0
PF05407.7	Rubella virus endopeptidase family.	0.49	20	0.77	33	7.62 × 10^− 5^
PF10895.3	Hydrophobic abundant protein (HAP) family.	0.48	18	0.77	27	2.28 × 10^− 4^
PF03866.8	RimK-like ATP-grasp domain family.	0.47	18	0.76	29	3.81 × 10^− 4^
PF05417.6	Protein of unknown function (DUF2715).	0.46	25	0.67	41	6.85 × 10^− 4^

PFAM ID is the accession number of the PFAM family, BLOSUM-FIRE score is the algorithm score, PID is the percentage identity score of the aligned sequences, dN/dS *sim* is the similarity of the dN/dS values (old FIRE algorithm score) and residues matched is the number of identical amino acid matched

**Fig. 10:**
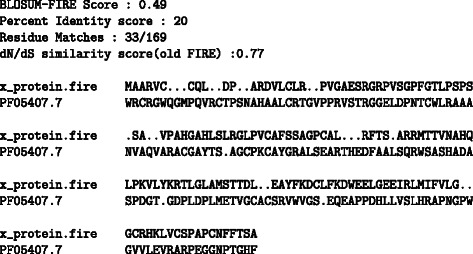
Top scoring alignment for HBx protein and PFAM family PF05407.7 corresponding to the rubella virus endopeptidase family. The number of aligned residues is relatively low

**Fig. 11:**
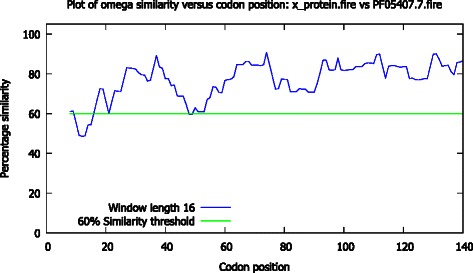
Plot of dN/dS similarity against codon position for the HBx and rubella endopeptidase protein family. The green line indicates the 60 % similarity threshold; the HBx and rubella endopeptidase protein dN/dS distributions are similar

**Fig. 12:**
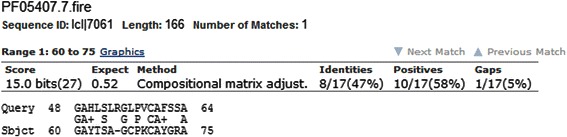
BLAST alignment for the HBx and rubella virus endopeptidase sequence. This was the only statistically significant alignment in addition to the transaction X family found by the BLAST algorithm

Since the association with the trans-activation X family is well known, we became interested in the similarity with the Rubella endopeptidase family as these domains have been found to play vital roles in viral replication. The BLOSUM-FIRE alignment for the HBx and rubella endopeptidase family is shown in Fig. 10. The codon similarity against codon position plot for the HBx and the rubella endopeptidase produced by the BLOSUM-FIRE algorithm is provided in Fig. 11. The plot reveals that the ω MLEs distribution for the two protein domains has high similarity even with a PID sore of 20 %. We also provide the BLAST alignment (Fig. 12) for the two proteins for comparison and the results show that there is a statistically significant region of similarity between the HBx and the rubella endopeptidase family. However, analysis of the BLAST results reveal a poor E-value when compared to the low E-values (generally an E value of 10^−4^ is used for inferring homologous relationships). The biological relevance of these results has not be established although the two proteins have vital roles in viral replication.

Some of inherent limitations of BLOSUM-FIRE approach to sequence alignment are the assumptions made when measuring positive selection under the M2a model using the CODEML program in the PAML suite. One of these assumptions is that for those sites under positive selection there have been numerous substitutions at that site across the phylogeny [11]; however, the selection pressure may vary across the lineages for example in HIV [44]. Additionally, it is assumed that the non-synonymous substitution rate value varies at codon sites while the same value is used for the synonymous substitution rate, this assumption can be violated in real data [45].

## Conclusion

This work provides evidence for the efficacy of an evolutionary rate based approach to sequence alignment; we also address the challenge of low specificity. We show that coupling evolutionary rates with conventional amino acid substitution matrices produces robust algorithms comparable in performance to conventional approaches to sequence alignment. We note that the approach has inherent limitations as methods and models of measuring the site by site evolutionary rate accurately still remain a challenging field. This work supports the hypothesis that proteins under similar selective pressures share similar functions. We provide a proof of concept that evolutionary rate profiles can be used as an alignment metric and that in certain cases at least the similarity of these evolutionary rate profiles can be used to infer domain functions. Additionally, we show that aligning sequences based on their evolutionary rate profiles could be used to extend the traditional alignment techniques in testing hypothesis in homology inference. The BLOSUM-FIRE software, user information and sample data files are freely available for download at http://www.bioinf.wits.ac.za/software/fire.
